# Histological pattern of tumor inflammation and stromal density correlate with patient demographics and immuno-oncologic transcriptional profile in oral squamous cell carcinoma

**DOI:** 10.3389/froh.2024.1408072

**Published:** 2024-06-06

**Authors:** Vasileios Ionas Theofilou, Ioana Ghita, Manar Elnaggar, Risa Chaisuparat, John C. Papadimitriou, Soren M. Bentzen, Donita Dyalram, Joshua E. Lubek, Robert A. Ord, Rania H. Younis

**Affiliations:** ^1^ Department of Oncology and Diagnostic Sciences, Division of Oral and Maxillofacial Pathology, University of Maryland School of Dentistry, Baltimore, MD, United States; ^2^ Department of Oral Pathology, Faculty of Dentistry, Chulalongkorn University, Bangkok, Thailand; ^3^ Department of Pathology, University of Maryland School of Medicine, Baltimore, MD, United States; ^4^ Division of Biostatistics and Bioinformatics, Department of Epidemiology and Public Health, University of Maryland School of Medicine, Baltimore, MD, United States; ^5^ Biostatistics Core, Institute of Clinical and Translational Research, University of Maryland, Baltimore, MD, United States; ^6^ Biostatistics Division, University of Maryland Marlene and Stewart Greenebaum Comprehensive Cancer Center, Baltimore, MD, United States; ^7^ Department of Oral and Maxillofacial Surgery, University of Maryland School of Dentistry, Baltimore, MD, United States; ^8^ Head and Neck Surgery Department of Oral and Maxillofacial Surgery, University of Maryland Marlene and Stewart Greenebaum Comprehensive Cancer Center, Baltimore, MD, United States; ^9^ Department of Oral Pathology, Faculty of Dentistry, Alexandria University, Alexandria, Egypt

**Keywords:** oral squamous cell carcinoma, tumor microenvironment, peritumoral stroma, cancer inflammation, oral cancer demographics, oncologic transcriptional profile

## Abstract

**Introduction:**

Oral squamous cell carcinoma (OSCC) is the most prevalent oral malignancy, with emerging interest in the characterization of its tumor microenvironment. Herein, we present a comprehensive histological analysis of OSCC stromal density and inflammation and their relationship with patient demographics, clinicopathologic features and immuno-oncologic signatures.

**Materials-methods:**

Eighty-seven completely excised OSCC tissues were prospectively collected and scored for histopathologic inflammatory subtypes [HIS]—inflamed (INF), immune-excluded (IE) and immune-desert (ID), peritumoral stromal inflammation (PTSI), and peritumoral stromal fibrosis (PTSF). Scoring of inflammation was complemented by Semaphorin 4D immunohistochemistry. NanoString differential gene expression (DGE) analysis was conducted for eight OSCC cases representative of the inflammatory and stromal subtypes and the demographic groups.

**Results:**

PTSF correlated with male gender (*p* = 0.0043), smoking (*p* = 0.0455), alcohol consumption (*p* = 0.0044), increased tumor size (*p* = 0.0054), and advanced stage (*p* = 0.002). On the contrary, PTSI occurred predominantly in females (*p* = 0.0105), non-drinkers (*p* = 0.0329), and small tumors (*p* = 0.0044). Transcriptionally, decreased cytokine signaling, and oncogenic pathway activation were observed in HIS-IE. Smokers and males displayed decreased global immune-cell levels and myeloid-cell predominance.

**Conclusion:**

Our work describes OSCC stromal and inflammatory phenotypes in correlation with distinct patient groups and DGE, highlighting the translational potential of characterizing the tumor microenvironment for optimal patient stratification.

## Introduction

Oral squamous cell carcinoma (OSCC) is the most common malignant neoplasm of the oral and maxillofacial region with almost 35,000 new cases projected in the United States in 2023 (1). Males have traditionally been more commonly involved than females (1), and classic risk factors, including smoking and alcohol, have been shown to induce oncogenic effects leading to carcinogenesis of the oral mucosa (2). However, recently the epidemiology of OSCC has been shifting to include patient profiles with non-traditional risk factors (females, non-smokers and non-drinkers), occasionally displaying distinct clinicopathologic features (e.g., common occurrence of proliferative verrucous leukoplakia in these demographic groups) (3), highlighting the heterogeneity of oral cancer etiology.

Despite the advances in understanding the molecular pathogenesis and initiating new treatment modalities, to this date the standard of care is surgical treatment in addition to chemotherapy and radiotherapy (4); with the overall 5-year survival being as low as 39% for distant metastatic disease (1). The need for more efficient therapeutic protocols for metastatic and recurrent disease, in addition to the increasing evidence of the deregulation of immune response during the progression of these neoplasms, necessitated the development and approval of immunotherapeutic agents for the treatment of head and neck tumors (5). Nevertheless, not all cancers respond to immunotherapy due to their distinct inflammatory and stromal phenotypes which render certain malignancies less prone to targeted therapies (6, 7). A particular emphasis has been given to the concepts of immune exclusion and non-inflamed tumors which correlate with decreased immunogenicity and reduced response to immunotherapy (7, 8).

The significant roles of the tumor microenvironment in OSCC at a microscopic level have been underestimated by the currently used WHO grading system, which assesses the degree of differentiation and does not emphasize other microscopic parameters of the tumor inflammation and stromal changes (9). Additionally, despite the introduction of parameters that affect prognosis (depth of invasion and extranodal extension) in the pathologic staging system (10), the tumor grade does not have any prognostic significance nor does it characterize response to therapeutic regimens (11). The necessity for potential future modifications in OSCC grading is supported by recent studies showing that non-inflamed (12) and fibrotic tumors with myofibroblastic proliferation (13, 14) seem to worsen the biologic behavior of OSCC.

Prior histopathologic and immunohistochemical studies have characterized in depth the tumor inflammation and stromal changes in various malignancies. This includes primarily assessing the “Immunoscore”, which characterizes the degree of inflammatory infiltration in cancer (15). Besides, the localization of inflammatory cells has also resulted into a subclassification of tumors into immune-inflamed (hot), immune-excluded (altered) and immune-desert (cold) tumors with effects on both prognosis and response to immunotherapy (12, 16–18). More specifically, besides the aforementioned resistance to immunomodulatory treatments during the absence of inflammatory response and the onset of immune exclusion (7, 8), non-inflamed histopathologic patterns correlate with unfavorable clinical outcome in cancer in general (7, 8) and in particular in OSCC [decreased survival in patients with immune-desert tumors (12)]. Most Immunoscores, characterize T cell inflammation (15, 19–21), while others have characterized the global levels of lymphocytic inflammation in H&E-stained tissue sections (12, 22). Our group has previously proposed a scoring system, to assess the pattern of tumor histological inflammatory subtype (HIS) using the expression of Semaphorin 4D (Sema4D) (16). Sema4D is a protein that has been shown to be expressed in T lymphocytes, activated B lymphocytes, dendritic cells (23) as well as tumor-associated macrophages (24), hence providing a more global characterization of OSCC inflammation (16).

Stromal changes have also been shown to affect the biologic behavior of cancer and its response to immunotherapy. More particularly the degree of desmoplasia assessed histologically as stromal fibrosis is a poor prognostic and predictive factor for OSCC (13, 14). Other authorities have also characterized the occurrence of a glycosaminoglycan-rich stroma, which is microscopically seen as a loose or myxoid stroma, however its biologic behavior has not been extensively assessed (11), with recent reports suggesting that an immature, myxoid stroma shares a poorer biological behavior (25).

Even though OSCC stroma and inflammation have been widely studied, their respective correlations with different patient profiles displaying distinct underlying pathogenesis, biologic behavior and response to treatment have not been investigated. Herein, we present a histopathologic and gene expression study of tissues retrieved from a well-characterized OSCC cohort. The OSCC microenvironment is described in detail, including the tumor inflammation and stromal changes via examination of H&E slides, complemented by Sema4D scoring and its correlation with the clinical characteristics, cancer risk factors and demographics is carried out as well. A transcriptomic analysis of a representative sample of OSCC stroma and inflammation and associated patient demographics using the NanoString technology is also presented. Collectively, our findings provide further insights into the roles of the tumor microenvironment in OSCC pathogenesis and suggest patient stratification schemes based on the tumor stroma and inflammation, that may open new avenues to facilitate the individualized management of OSCC.

## Materials-methods

### Patient samples

**Figure 1:**
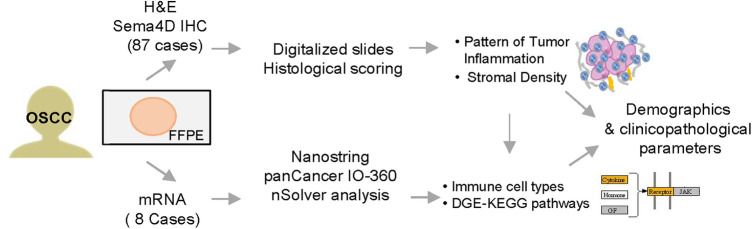
Experimental workflow. Tissues from 87 patients with oral squamous cell carcinoma (OSCC) were prospectively included in the study. Histological scoring and transcriptional findings were analyzed in correlation to patients' demographics and clinico-pathological characteristics. H&E, hematoxylin and eosin stain; IHC, immunohistochemistry; Sema4D, Semaphorin 4D; DGE, differential gene expression.

Head and neck squamous cell carcinoma (HNSCC) samples from 104 patients were collected prospectively upon patient informed consent and under institutional review board (IRB) approved protocol at the University of Maryland School of Medicine (UMSOM) (HP-00073603) (16). The patients/participants provided their written informed consent to participate in this study. The study was performed in accordance with the Declaration of Helsinki. The inclusion criteria for the current study were primary tumors, biopsy confirmed as OSCC of the oral and mobile tongue, and independently decided for complete surgical excision as the initial line of treatment. Tissue collected during surgery were fixed in 10% formalin and embedded in paraffin. The tissue-based experimental approaches used in our studies are presented in subsequent sections and are summarized in Figure 1.

### Histopathologic scoring of OSCC inflammation and stromal density

A categorization of the tumor stroma and inflammation was performed subjectively on hematoxylin and eosin (H&E) and Semaphorin 4D (Sema4D) immunohistochemistry (IHC) which our group has previously standardized as a complementary method to score global immune cell infiltration (16, 24). The tumor sections were scanned using Aperio Scanscope and scored by three surgical pathologists (RY, RC and VT). The histopathologic scoring evaluated the pattern of histological inflammatory subtype (HIS), which included HIS inflamed (HIS-INF), immune excluded (HIS-IE) and immune deserted (HIS-ID). Other included scoring criteria were peritumoral stromal inflammation (PTSI), peritumoral stromal fibrosis (PTSF), diffuse stromal fibrosis (SF), and fibromyxoid (FMX) stroma.

### Nanostring gene expression analysis of OSCC

For the differential gene expression (DGE) analysis, 8 OSSC cases representative of the stromal and inflammatory phenotypes were mapped to include the tumor and 1–3 mm peritumoral stromal margin. The tumor was scrapped off the slides and total RNA was extracted using Rneasy FFPE kit (Qiagen). RNA quality control (QC), followed by hybridization according to the nCounter® PanCancer Immune Profiling Panel of NanoString was performed as previously described (1). The extracted raw data from the NanoString analysis are available and can be found here: https://www.ncbi.nlm.nih.gov/geo/query/acc.cgi?acc=GSE220863. Basic and advanced DGE analysis in the nSolver 4.0 software were performed to characterize the immuno-oncologic profile of the scored HIS subtypes, PTSI, PTSF, and SF with patient demographics. Briefly, raw data were imported and annotated based on HIS (3 cases of HIS-INF OSCC vs. 3 cases of HIS-IE), PTSI (4 cases vs. 4 baseline OSCC cases with no PTSI), PTSF (3 cases vs. baseline 5 cases of tumors without PTSF), gender (4 females vs. 4 baseline males) and smoking habits (3 non-smokers vs. 5 baseline smokers). Kegg pathways, including pathways in cancer and cytokine pathways, in addition to individual immune cell type characterization were carried out.

### Statistical analysis

For the investigation of possible associations of OSCC stroma with clinical characteristics and demographics, non-parametric statistical tests were used. To explore the roles of demographics and risk factors in generating distinct stromal and inflammatory phenotypes, the age, gender, smoking and alcohol consumption as well as prior chemo- or radiotherapy were used as independent variables and HIS (INF vs. IE and ID), PTSI, PTSF, SF and FMX were used as dependent variables. For age correlations (continuous variable) simple logistic regression was used, while for categorical variables (gender, smoking and alcohol) Fisher's exact test was used. On the other hand, to correlate the tumor stroma with other clinicopathologic findings the HIS, PTSI, PTSF, SF and FMX were used as independent variables and tumor size, stage, and histopathologic grade as dependent variables. Mann-Whitney test was used in correlations with size and Fisher's exact test for the remaining categorical variables. Statistically significant results were considered those with *p* < 0.05. Statistical correlations of the DGE analysis were conducted with the nSolver 4.0 software with statistical significance of *p* < 0.05.

## Results

### Scoring of the pattern of tumor inflammation and stromal density

HNSCC samples from 104 patients were prospectively collected. Eight cases of oropharyngeal and base of tongue were excluded from the current study. Tumor tissue was examined on H&E. Seven cases were excluded due to lack of peritumoral stroma, two cases were excluded due to lack of tumor tissue in surgical specimen. The available clinicopathologic characteristics and clinical risk factors described in the current work are focused on the 87 OSCC patients presented in Supplementary Table S1. Among the included cases, if individual clinical or histopathologic parameters were not available or could not be evaluated, statistical analysis was conducted only for the available parameters.

**Figure 2:**
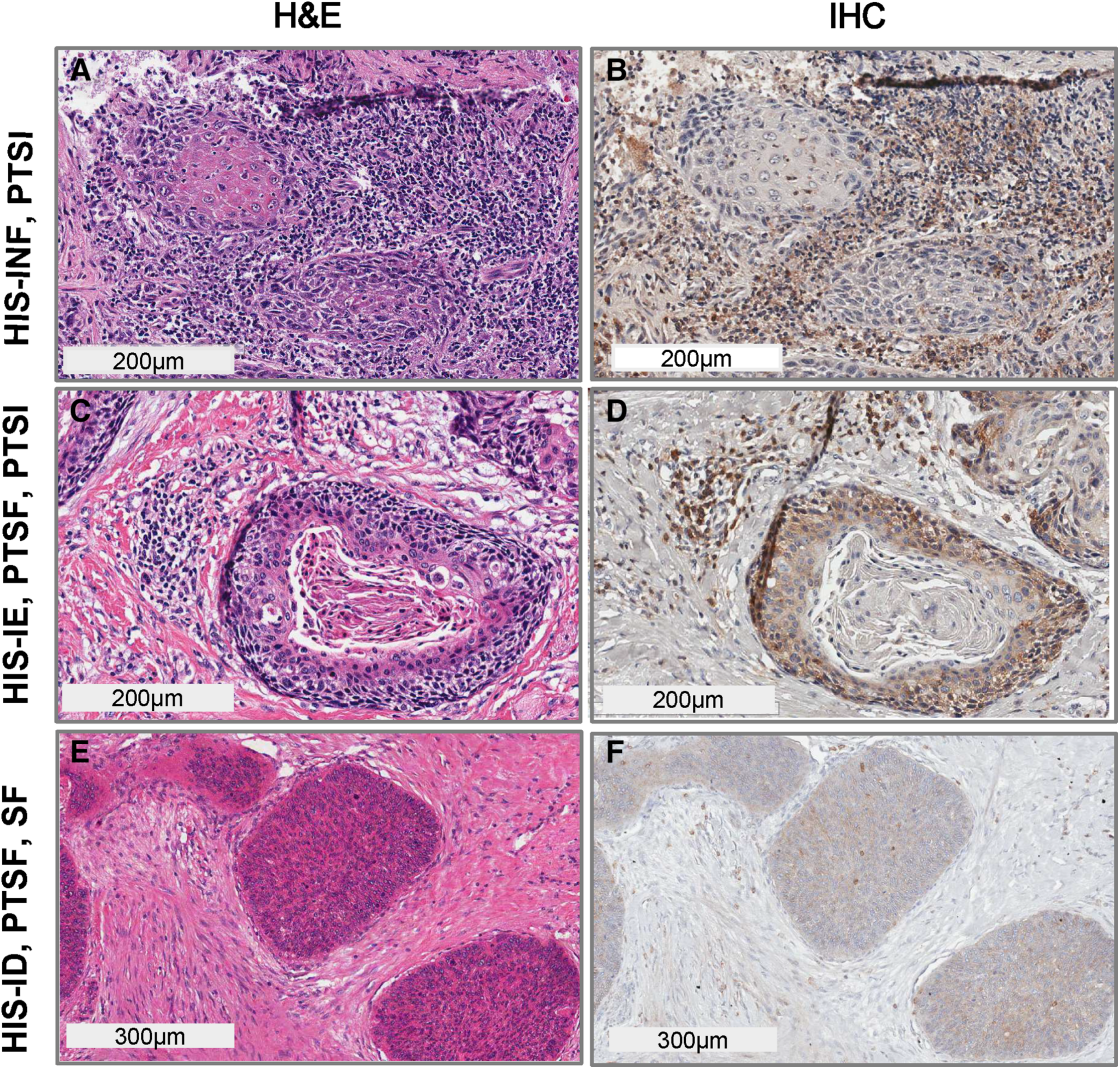
Histological scoring for diverse inflammatory and stromal density findings in OSCC. HIS subtype, PTSI, PTSF and SF. (**A**) H&E stain and (**B**) Sema4D IHC, of OSCC denoting HIS-INF phenotype with PTSI high; (**C**) H&E stain, (**D**) Sema4D IHC, denoting HIS-IE with PTSF-high, and PTSI high; (**E**) H&E stain, (**F**) Sema4D IHC stain of HIS-ID, PTSF high, and SF high. HIS, histological inflammatory subtype; INF, inflamed; IE, immune excluded; ID, immune deserted; PTSI, peritumoral stromal inflammation; PTSF, peritumoral stromal fibrosis; SF, stromal fibrosis.

We have previously assessed the patterns of HIS, using H&E and Sema4D stained sections. Here we wanted to further characterize the tumor inflammation and stromal density through scoring for several other variables that included the peritumoral stromal inflammation (PTSI), peritumoral stromal fibrosis (PTSF) and generalized stromal fibrosis (SF) or fibromyxoid stroma (FMX). For the current OSCC focused cohort, the HIS-INF (histologically inflamed), identified as OSCCs with prominent inflammatory cell infiltration into the tumor islands (≥5 cells per moderately sized tumor island ∼size range), occupying the majority of the tumor (Figures 2A,B). The HIS-IE had inflammatory cells infiltration at the tumor invasive front or in areas between the OSCC islands, however, the inflammatory cells did not extend into the tumor islands (<5 cells within a tumor island of moderate size) that can be “excluded” by a thin zone of fibrous or fibromyxoid connective tissue surrounding the tumor island (Figures 2C,D) (Supplementary Image S1). The immune-desert tumors (HIS-ID or “cold” tumors, (Figures 2E,F) were partially or completely deserted of the inflammatory infiltration with only focal areas of inflammation if any, that would be mainly located in the tumor invasive front.

Heterogeneous tumors in which HIS-IE or HIS-ID were combined with areas of HIS-INF were scored as IE or ID if the according changes were observed in more than 25% of the tumor surface. HIS-INF was seen more commonly compared to HIS-IE or HIS-ID, [49 HIS-INF (56%), 31 HIS-IE (36%) and 7 HIS-ID (8%)].

The peritumoral stromal inflammation (PTSI) was scored independent of inflammatory cell extension into the tumor islands (Figures 2A–D) and considered whether the inflammatory cells are infiltrating the areas around the tumor islands accounted for more than 50% of the tumor tissue. PTSI (Figures 2A–D) represents the presence of prominent inflammatory infiltration in peritumoral locations which may be observed in either HIS-INF (Figures 2A,B) or in a subset of HIS-IE (Figures 2C,D). We also characterized as PTSF (Figures 2C–F), the presence of a desmoplastic, fibrotic zone of connective tissue localized directly around or juxtaposed to tumor islands in more than 50% of the OSCC surface area.

The overall findings of the tumor stroma, describing the occurrence of diffuse stromal fibrosis (SF), occupying the majority of connective tissue around the tumor and not exclusively the peritumoral area was also analyzed. Additionally, we scored for the frequency of fibromyxoid stroma (FMX) which was described as a loose, myxomatous or mucinous stromal changes diffusely occupying the connective tissue or exclusively in the peritumoral area, accounting for more than 50% of OSCC surface (Supplementary Image S1A–C). It is worth noting that the PTSF is characterized by a peritumoral dense, desmoplastic stroma which may or may not be combined with SF (Figures 2C–F).

### HIS subtype correlates with patient demographics and tumor characteristics

**Figure 3:**
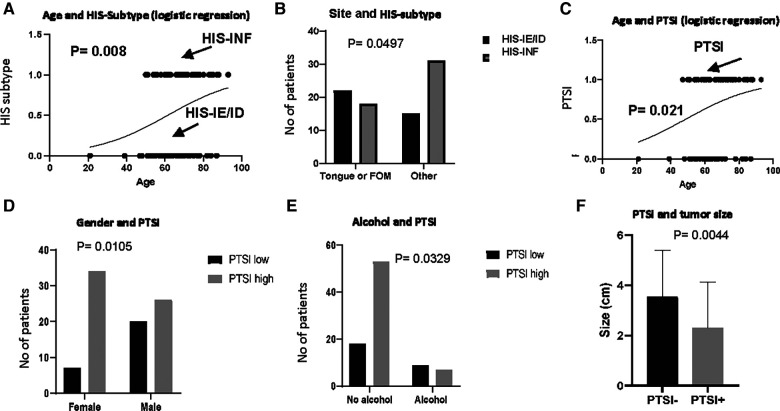
HIS subtype and PTSI in correlation to patient demographics and clinico-pathologic characteristics. HIS subtype in correlation to (**A**) age and (**B**) site of involvement. PTSI in correlation to (**C**) age, (**D**), gender, (**E**) alcohol drinking and (**F**) size of the tumor.

We subsequently investigated whether the pattern of global inflammation correlates with patient clinical characteristics and demographics, concluding that non-inflamed (HIS-IE or HIS-ID) tumors were observed in patients of almost one-decade younger age group compared with HIS-INF (*p* = 0.008) (Table 1, Figure 3A). Additionally, the tongue as well as floor of the mouth, which are considered high-risk oral cancer sites, were involved more commonly in HIS-IE/ID OSCC compared with HIS-INF (*p* = 0.0497) (Table 1, Figure 3B).

**Table 1:** Correlations between the histological variables (HIS, PTSI and PTSF) and patient characteristics.

Characteristics	HIS		*p* Value	PTSI		*p*-Value	PTSF		*p*-Value
	INF (%)	IE/ID (%)		Yes (%)	No (%)		Yes (%)	No (%)	
Age—mean (median)	69.44 (69)	62.84 (62.5)	0.008**	68.5 (68)	62.3 (63)	0.021*	65.7 (65)	67.4 (67)	0.519
Gender									
Female	27 (66)	14 (34)	0.129	34 (83)	7 (17)	0.011*	10 (25)	30 (75)	0.004**
Male	22 (48)	24 (52)		26 (57)	20 (43)		26 (57)	20 (43)	
Smoking									
No	31 (61)	20 (39)	0.382	39 (76)	12 (24)	0.010	16 (32)	34 (68)	0.046*
Yes	18 (50)	18 (50)		21 (58)	15 (42)		20 (56)	16 (44)	
Alcohol									
No	42 (59)	29 (41)	0.279	53 (75)	18 (25)	0.033*	24 (34)	46 (66)	0.004**
Yes	7 (44)	9 (56)		7 (44)	9 (56)		12 (75)	4 (25)	
Prior chemotherapy									
No	46 (59)	32 (41)	0.1705	56 (72)	22 (28)	0.1287	31 (40)	46 (60)	0.4819
Yes	3 (33)	6 (67)		4 (44)	5 (56)		5 (56)	4 (44)	
Prior radiotherapy									
No	40 (59)	28 (41)	0.4377	49 (72)	19 (28)	0.2692	26 (39)	41 (61)	0.3038
Yes	9 (47)	10 (53)		11 (58)	8 (42)		10 (53)	9 (47)	
Site of involvement^a^									
Tongue or floor of mouth	18 (45)	22 (55)	0.050	25 (63)	15 (38)	0.352	15 (38)	24 (62)	0.519
Other	31 (67)	15 (33)		34 (74)	12 (26)		21 (46)	25 (54)	
Size (median in cm)	1.95	2.55	0.101	2	4	0.004**	3	1.7	0.005**
Stage									
I or II	20 (56)	16 (44)	>0.99	27 (75)	9 (25)	0.353	8 (22)	28 (78)	0.002**
III or IV	29 (57)	22 (43)		33 (65)	18 (35)		28 (56)	22 (44)	
Grade									
I	26 (60)	17 (40)	0.518	33 (77)	10 (23)	0.165	14 (33)	28 (67)	0.138
II or III	23 (52)	21 (48)		27 (61)	17 (39)		22 (50)	22 (50)	

Percentages of HIS, PTSI and PTSF per patient group are rounded-up to the nearest ones place.

^a^ Detailed number of samples: tongue (35), mandible/retromolar (21), maxilla/palate (9), buccal mucosa/lip (9), gingiva with no alveolar bone involvement (6), floor of mouth (5), simultaneous maxillary & mandibular involvement (1).

*p value <0.05, >0.01. **p value ≤0.01.

### PTSI correlates with demographics and better tumor behavior of OSCC

Besides characterizing the pattern of inflammatory infiltration into the tumor island, we scored for additional histological criteria depending on the presence or absence of inflammation in peritumoral locations which was observed in both the HIS-INF (Figures 2A,B) and in certain HIS-IE tumors (Figures 2C,D). PTSI was seen in the majority of tumors (60 out of 87 tumors) and displayed significant correlations with distinct patient profile. More specifically, PTSI was observed in older patients (median age of 68 years) compared with the PTSI negative group (median age of 63 years, *p* = 0.021, Table 1, Figure 3C). In addition, PTSI correlated with female patients (*p* = 0.0105, Table 1, Figure 3D) and non-alcohol drinkers (*p* = 0.0329, Table 1, Figure 3E). Correlating with patient demographics, PTSI showed indications of a better biological behavior due to its occurrence in smaller tumors (2 cm median size vs. 4 cm in no PTSI, *p* = 0.0044, Table 1, Figure 3F).

### Stromal density correlates with demographics and poor tumor behavior of OSCC

After evaluating OSCC inflammation (by characterizing the HIS and PTSI) we also described stromal characteristics of the tumor microenvironment. First of all, we characterized the presence of PTSF (i.e., the occurrence of desmoplastic rim located at the periphery of the tumor islands, Figures 2C–F). Additionally, as already described we assessed stromal changes that were observed diffusely in the connective tissue including SF and FMX stroma (Supplementary Images S1A–C).

**Figure 4:**
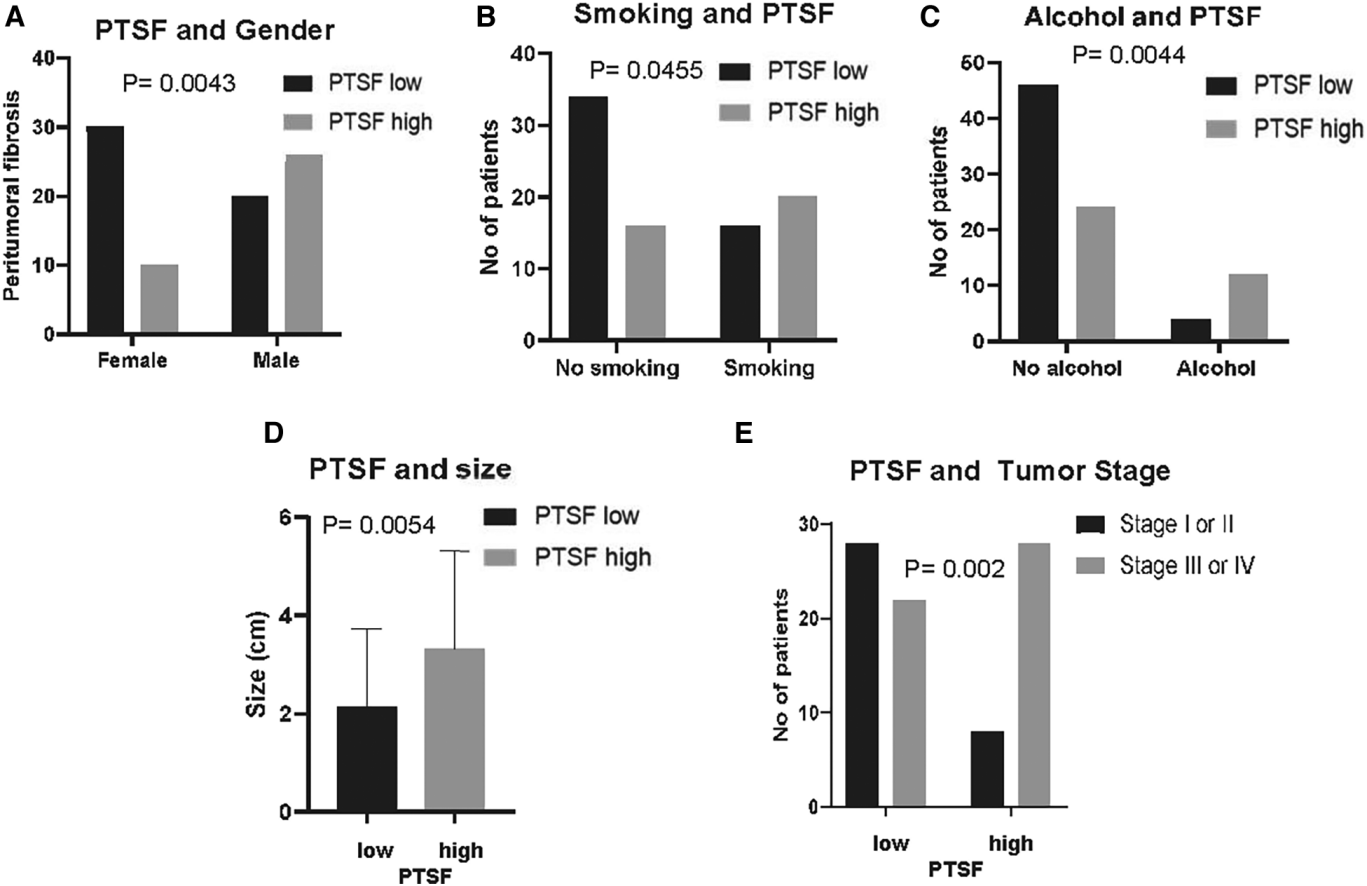
PTSF in correlation to patient demographics and clinic-pathological characteristics. PTSF in correlation to (**A**) gender, (**B**) smoking, (**C**) alcohol drinking, (**D**) tumor size, and (**E**) tumor stage.

PTSF was observed in 36 cases compared with 50 with no PTSF and interestingly correlated with patient profile, including male gender (*p* = 0.0043) as well as smoking (*p* = 0.0455) and drinking (*p* = 0.0044) habits. Additionally, OSCC size was increased (*p* = 0.0054), and stage was advanced (*p* = 0.002), which collectively indicate that PTSF correlates with a poorer biological behavior (Table 1, Figure 4). Diffuse SF also showed similar correlations with the tumor size (*p* = 0.0114), and stage (*p* = 0.0004) in addition to history of radiotherapy (*p* = 0.0081) (Supplementary Table S2). No statistical correlations between FMX and patient characteristics were observed (data not shown).

### Immuno-oncologic analysis of OSCC stromal and inflammatory phenotypes

**Figure 5:**
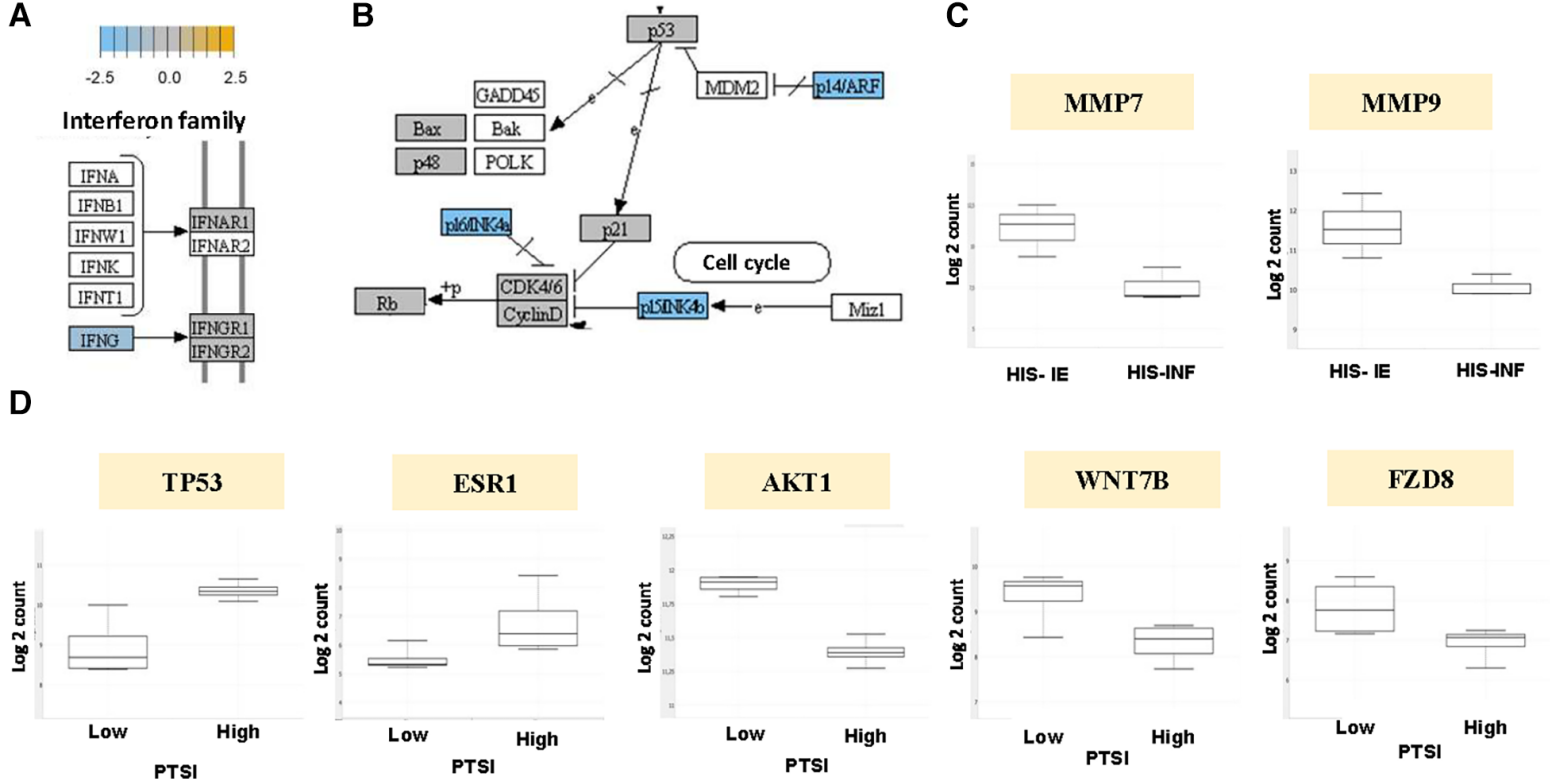
Kegg pathway analysis of the HIS subtype and PTSI. DGE depicted in HIS-IE OSCC relative to baseline of HIS-INF phenotype for (**A**) IFN-γ, and (**B**) negative cell cycle regulators P16INK4a, P14ARF, P15INK4b. (**C**) DGE of MMP-7 and MMP-9 for HIS-IE relative to HIS-INF. (**D**) DGE of TP53, ESR1, AKT1, WNT7B and FZD8 for PTSI-high, relative to baseline of PTSI-low, OSCC.

To investigate whether the distinct roles of the inflammatory and stromal phenotypes correlate with gene expression profile, we conducted a pilot DGE analysis utilizing the immuno-oncologic profiling panel of NanoString technology. Our first goal was to evaluate triplicates of HIS-INF compared with HIS-IE OSCC. Our analysis showed the HIS-IE OSCC to have differentially decreased IFN-γ expression compared to the HIS-INF and significant increase in the CNTFR (ciliary neurotrophic factor receptor), with differential increase in CD19, CD38, and GZM in the HIS-INF (Figure 5A) (Supplementary Image 1D). Additionally, the DGE profile of the Kegg pathways “pathways in cancer” showed downregulation of negative regulators of the cell cycle in the HIS-IE (P16INK4a, P14ARF and P15INK4b, Figure 5B). Fas-L was also downregulated as well as Cytokines-cytokine receptor interaction. Upregulation of PTEN and PI3K, Hey, PPARγ and PPFP. Finally, matrix metalloproteinases, specifically MMP-7 and MMP-9 mRNA levels were shown to be significantly upregulated in the HIS-IE (Figure 5C, Supplementary Image S2).

Our second goal to further characterize the underlying molecular manifestations of tumor inflammation was to set as variable the PTSI (4 cases of PTSI vs. 4 control OSCC cases). PTSI correlated with tumor suppressing signaling events, specifically upregulation of p53. In addition, differential overexpression of the estrogen receptor (ESR) signaling was noted in the PTSI high group (Figure 5D, Supplementary Image S3). A decreased expression of members of the oncogenic pathways including the PKB/Akt and Wnt/Frizzled pathway (WNT7B and FZD8) was observed (Figure 5D). Tumors with PTSF showed upregulation of HIF-β, Delta upstream of Notch, ET1 upstream of GPCR, and PKC upstream of Raf and downregulation of VEGF, Cyclin D1, CDK4/6, FADD downstream of FasL and Jagged upstream of Notch (Supplementary Image S4).

**Figure 6:**
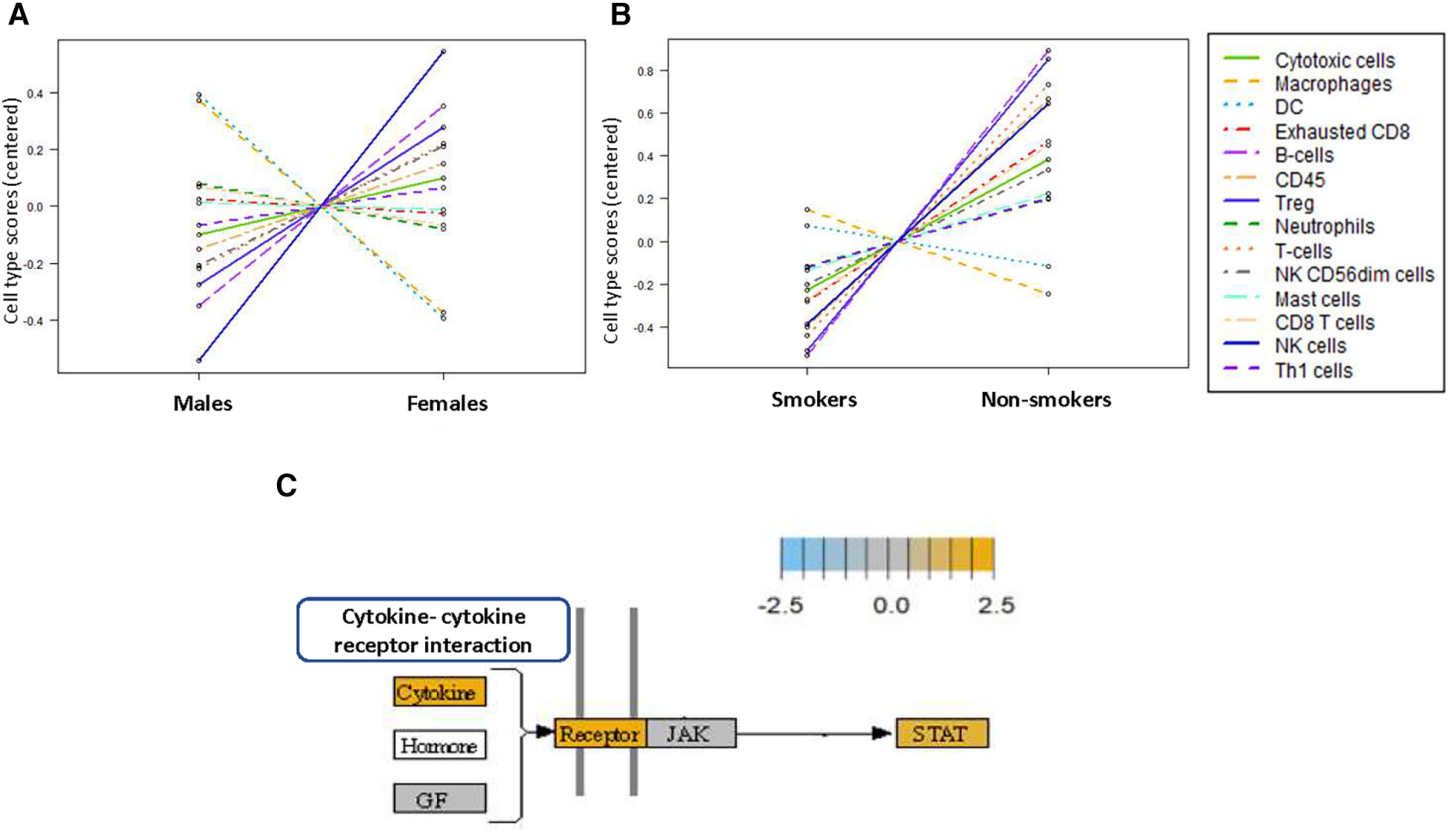
Distinct immune cell type in OSCC demographic subgroups. Trend plots of the immune cell type in OSCC according to (**A**) gender and (**B**) smoking habits. (**C**) Kegg pathway cytokine signaling analysis of non-smokers OSCC relative to baseline of smokers.

To further highlight the possible correlation between the tumor microenvironment and patient demographics, we also annotated for the gender (4 females and 4 males) and smoking habits (5 smokers and 3 non-smokers). Cell type sorting indicated that female gender and non-smoking habits correlated with increased global levels of immune cells, and predominance of lymphoid lineage of cells; in contrast to males and smokers showing predisposition for immune cells of myeloid origin. Specifically, males showed differentially higher population of dendritic cells and macrophages, followed by neutrophils, exhausted CD8 cells and mast cells. Females were differentially higher in NK cells, B cells, Tregs, and cytotoxic T cells and T-helper 1. Non-smokers showed higher B cells, T cells, NK cells, exhausted CD8 cells, and cytotoxic cells, followed by mast cells. Dendritic cells and macrophages were higher in the smokers group. (Figures 6A,B). Kegg pathways for cytokine signaling showed increased signaling both the upstream and downstream molecules with increased mRNA levels for cytokines, cytokine receptors and STAT molecules in non-smokers relative to smokers' group. (Figure 6C, Supplementary Image S5).

## Discussion

This is, to the best of our knowledge, the first study to investigate potential associations between the tumor microenvironment and patient demographics in OSCC. The need for personalized management of cancer has strengthened the concept for addressing interpatient tumor heterogeneity and subclassifying disease depending on parameters that affect prognosis, predict susceptibility to immunotherapy and consequently help achieve optimal therapy for each individual case (26). In this notion, our findings specifically suggest that a subset of patients, i.e., female patients, non-smokers and non-drinkers, are displaying inflamed tumors, implicating a phenotype that can be a favorable candidate for immunotherapeutic agents (7).

Hormonal responses have previously been implicated in inducing inflammatory changes (27) as well as affecting inflammation in oral cancer (28). To further support this, our gene expression analysis indicated that increased estrogen receptor signaling was observed in tumors with high PTSI. That was in concordance with the demographic correlation with female gender, nonsmokers, and nondrinkers, demonstrating low PTSF. Interestingly, the effect of cigarette smoking in causing global decrease of tumor inflammation and interferon-γ signaling has been previously supported by sequencing data retrieved from the Cancer Genome Atlas (29), while alcohol has also been implicated in decreased tumor inflammation (30). The potential management of smokers and alcohol consumers with treatment modalities that increase the degree of inflammatory infiltration has also been reported (30). Collectively, the data presented herein underpin the need for a detailed characterization of stromal and inflammatory changes in OSCC to facilitate the prognostication and to improve the quality of life in different patient groups.

Our analysis also showed supporting findings of a possible prognostic significance of PTSI and PTSF. More specifically, PTSI correlated with decreased size and PTSF with larger tumors and higher stages of OSCC. The poor biologic behavior of immune-desert (12) as well as fibrotic (13) OSCCs and tumors with high stroma-tumor ratio (14) have been previously described, however, evaluating the prognostic significance of PTSI and PTSF after the prospective evaluation of our cohort is one of our future directions. Interestingly, a subset of female non-smoker patients who suffer from proliferative verrucous leukoplakia, developed cancers of better biologic behavior (31), similar to that described in studies of inflamed tumors (12). On the contrary, oral cancer in smokers, who we showed to display more fibrotic changes, exhibit worse prognosis (32).

Of note, none of the histopathologic parameters of the stroma and inflammation correlated with the tumor grade. The necessity for incorporation of histopathologic findings observed in the tumor stroma including different immune-scores measuring the T-cell infiltrate, as well as describing the degree of stromal desmoplasia has been previously emphasized (11). The histopathologic parameters presented herein (HIS, PTSI, and PTSF) can be measured exclusively via examination of H&E stained-slides, hence may be applied easily in everyday practice, ideally complemented with a pan-immune cell immunohistochemical marker (e.g., CD45 or Sema4D). Additionally, we were the first to substantially highlight the stromal findings in peritumoral locations (PTSI and PTSF) in OSCC instead of scoring inflammation and fibrosis diffusely in the tumor stroma. Notably, PTSF and PTSI showed more significant correlations with patient demographics compared with the global levels of inflammation and fibrosis. Provided that the prognostic significance of the PTSF and PTSI is validated, they could serve as independent parameters in future grading systems.

Our gene expression analysis using the Nanostring technology also complemented our findings and supported the correlations of the tumor microenvironment with distinct phenotypes and biologic behavior. The presence of inflammation at a microscopic level (HIS-INF and PTSI) was validated at a transcriptional level by increased cytokine signaling, while as previously highlighted, immune-excluded tumors showed decreased interferon signaling (7). Additionally, PTSI and HIS-INF showed relatively decreased mRNA levels of classic oncogenic markers including members of the Wnt/β catenin pathway and PI3K pathway. These signaling mechanisms have also been implicated in reducing T cell infiltration in other types of malignancies including lung cancer, in accordance with our data (33). Loss of TP53 gene function, a classic tumor suppressor has also been previously shown to decrease cancer-associated T cell inflammation (33), similar to our findings showing it increased in PTSI phenotype. Additionally, the implication of various other “hallmarks” of cancer, including the induction of angiogenesis and downregulation of growth suppressors (34) in non-inflamed tumors has previously been emphasized (8) and was also demonstrated in our cohort. On the contrary, using annotations for PTSF (Supplementary Image S5) and SF (data not shown), these parameters correlated with tumor suppressive events including downregulation of cell-cycle promoter Cyclin D1 and angiogenic factor VEGF (8), yet they showed to upregulate HIFβ implicated in tumor hypoxia (35), PKC involved in calcium signaling and cellular contractility (36), and ET1, the aberrant expression of which was shown to promote tumorigenesis (37). These results suggest tumor dependency on alternate pathways, reflecting different distinct phenotypes and further support the existing literature on the pathogenesis of stromal alterations in cancer. More specifically, oncogenic pathways originating from tumor cells, local immunomodulatory signals and mechanical stimuli have the capacity to convert stromal fibroblasts to cancer associated fibroblasts that have distinct morphologic features (increased contractility with aSMA expression), display unique metabolic reprogramming and extracellular matrix deposition (histologically ranging from fibromyxoid stroma to fibrosis) (38).

Finally, our mRNA analysis also presents similar results indicating that gender and use of carcinogens affect the global levels and relative distributions of immune cells. Females and non-smokers showed increased numbers of inflammatory cells, and higher abundance of cells of lymphoid lineage. In the contrary, males and smokers displayed decreased levels of inflammatory cells and predominance of cells of myeloid lineage. The effects of smoking in implicating immune cells of myeloid origin, including inducing macrophage polarization to an M2 phenotype in early carcinogenesis (39) and generating fibroblast-induced macrophage migration (40) has been emphasized. Additionally, the increase of antigen presentation in fibrotic and “cold” tumors has also been highlighted (7).

## Conclusion

**Figure 7:**
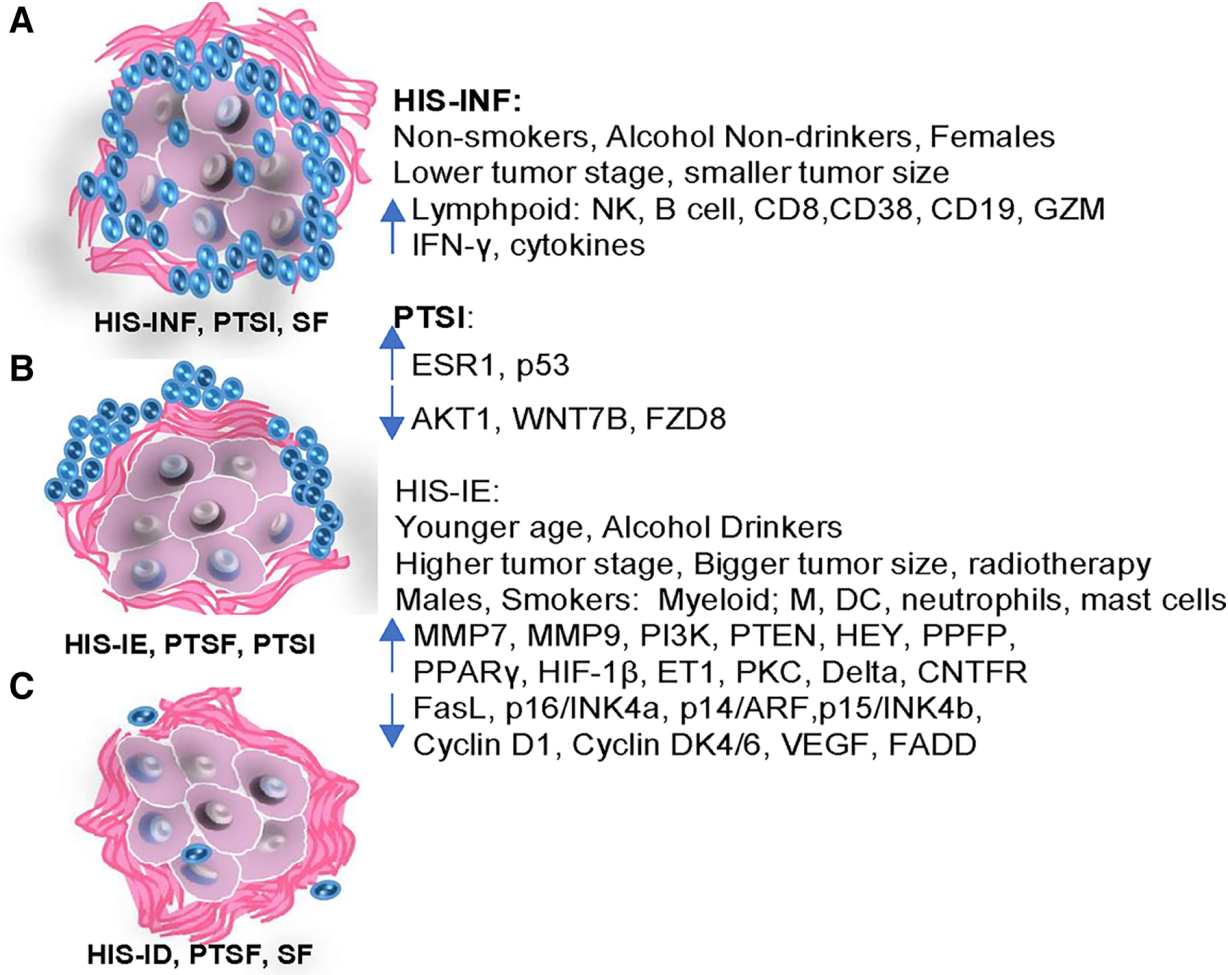
Diagrammatic illustration of the differential demographic and clinicopathological features correlative to the immuno-oncologic profile of HIS-INF and PTSI, compared to the HIS-IE, PTSF and HIS-ID.

This is the first study to characterize the tumor microenvironment with regards to its correlation to the OSCC patient demographics and transcriptional immuno-oncologic profile (Figure 7). In particular, females, non-smokers, and non-drinkers seem to develop tumors with increased inflammation and better biologic behavior, while males, smokers and alcohol consumers correlate with non-inflamed tumors with fibrotic changes, and a possible diminished biological behavior. New histopathologic parameters and scoring methods (HIS, PTSI and PTSF) have been proposed that might effectively complement future OSCC grading systems for better patient stratification.

## Data Availability

The datasets presented in this study can be found in online repositories (https://www.ncbi.nlm.nih.gov/geo/query/acc.cgi?acc=GSE220863).

## Supplementary material

Supplementary Image 1 Stromal Density Characterizing Parameters. (**A,B**) SF was described as the presence of diffusely desmoplastic stroma in the areas of the tumor independent of the HIS subtype. (**C**) FMX stroma was the presence of loose, myxoid connective tissue adjacent to the tumor nests. (**D**) DGE analysis of HIS-INF relative to base line of HIS-IE. HIS, histologic inflammatory subtype; ID, immune deserted; INF, inflamed; IE, immune excluded; SF, stromal fibrosis; FMX, fibromyxoid; DGE, differential gene expression.

Supplementary Image 2 Kegg pathway analysis for “Pathways in Cancer” in HIS-IE vs. baseline of HIS-INF OSCC.

Supplementary Image 3 Kegg pathway analysis for “Pathways in cancer” using annotations of PTSI high vs. baseline of PTSI low.

Supplementary Image 4 Kegg pathway analysis for “Pathways in cancer” signaling using annotations of PTSF high relative to baseline of PTSF low.

Supplementary Image 5 Kegg pathway analysis for cytokine/cytokine receptor signaling using non-smokers vs. baseline of smokers’ annotations.
