# Supplementary material for: Histological pattern of tumor inflammation and stromal density correlate with patient demographics and immuno-oncologic transcriptional profile in oral squamous cell carcinoma
Source: Front Oral Health. 2024 Jun 6;5:1408072. doi: 10.3389/froh.2024.1408072 (PMC11187265; doi:10.3389/froh.2024.1408072)
Supplement: Supplementary file 1 [file Table1.docx]

**Supplementary Table 1:** Clinical characteristics and demographics of 87 OSCC patients.

| **Characteristics** | **Number (%)** |
| --- | --- |
| **Age - mean (median)** | 66.56 (67) |
| **Gender** |  |
| Female | 41 (47) |
| Male | 46 (53) |
| **Smoking** |  |
| No | 51 (59) |
| Yes | 36 (41) |
| **Alcohol** |  |
| No | 16 (18) |
| Yes | 71 (82) |
| **Prior Chemotherapy** |  |
| No | 78 (90) |
| Yes | 9 (10) |
| **Prior Radiotherapy** |  |
| No | 68 (78) |
| Yes | 19 (22) |
| **Site of Involvement** |  |
| Tongue or floor of mouth | 40 (47) |
| Other^*^ | 46 (53) |
| **Size (median)** | 2.2 cm |
| **Stage** |  |
| I or II | 36 (41) |
| III or IV | 51 (59) |
| **Grade** |  |
| I | 43 (49) |
| II or III | 44 (51) |

***** other, refers to buccal mucosa, palatal mucosa or gingiva.
