# Supplementary material for: Histological pattern of tumor inflammation and stromal density correlate with patient demographics and immuno-oncologic transcriptional profile in oral squamous cell carcinoma
Source: Front Oral Health. 2024 Jun 6;5:1408072. doi: 10.3389/froh.2024.1408072 (PMC11187265; doi:10.3389/froh.2024.1408072)
Supplement: Supplementary file 2 [file Table2.docx]

**Supplementary Table 2:** Statistical correlations between SF and patient characteristics

| **Characteristics** | **SF** | | **P Value** | |
| --- | --- | --- | --- | --- |
|  | **Yes** | **No** | |  |
| **Age - mean (median)** | 67.4 (69) | 66.0 (63) | | 0.5948 |
| **Gender** |  |  | |  |
| Female | 15 (38) | 25 (63) | | 0.1979 |
| Male | 24 (52) | 22 (48) | |  |
| **Smoking** |  |  | |  |
| No | 22 (44) | 28 (56) | | 0.8281 |
| Yes | 17 (47) | 19 (53) | |  |
| **Alcohol** |  |  | |  |
| No | 29 (41) | 41 (59) | | 0.1667 |
| Yes | 10 (63) | 6 (38) | |  |
| **Prior Chemotherapy** |  |  | |  |
| No | 33 (43) | 44 (57) | | 0.2889 |
| Yes | 6 (67) | 3 (33) | |  |
| **Prior Radiotherapy** |  |  | |  |
| No | 25 (37) | 42(63) | | 0.0081** |
| Yes | 14 (74) | 5 (26) | |  |
| **Site of Involvement** |  |  | |  |
| Tongue or floor of mouth | 17 (44) | 22 (56) | | >0.999 |
| Other | 21 (47) | 25 (53) | |  |
| **Size (median)** | 2.9 cm | 1.7 cm | | 0.0114* |
| **Stage** |  |  | |  |
| I or II | 8 (22) | 28 (78) | | 0.0004** |
| III or IV | 31 (62) | 19 (38) | |  |
| **Grade** |  |  | |  |
| I | 16 (38) | 26 (62) | | 0.2019 |
| II or III | 23 (52) | 21 (48) | |  |

SF; stromal fibrosis
