# Supplementary figures and images for: Histological pattern of tumor inflammation and stromal density correlate with patient demographics and immuno-oncologic transcriptional profile in oral squamous cell carcinoma
Source: Front Oral Health. 2024 Jun 6;5:1408072. doi: 10.3389/froh.2024.1408072 (PMC11187265; doi:10.3389/froh.2024.1408072)

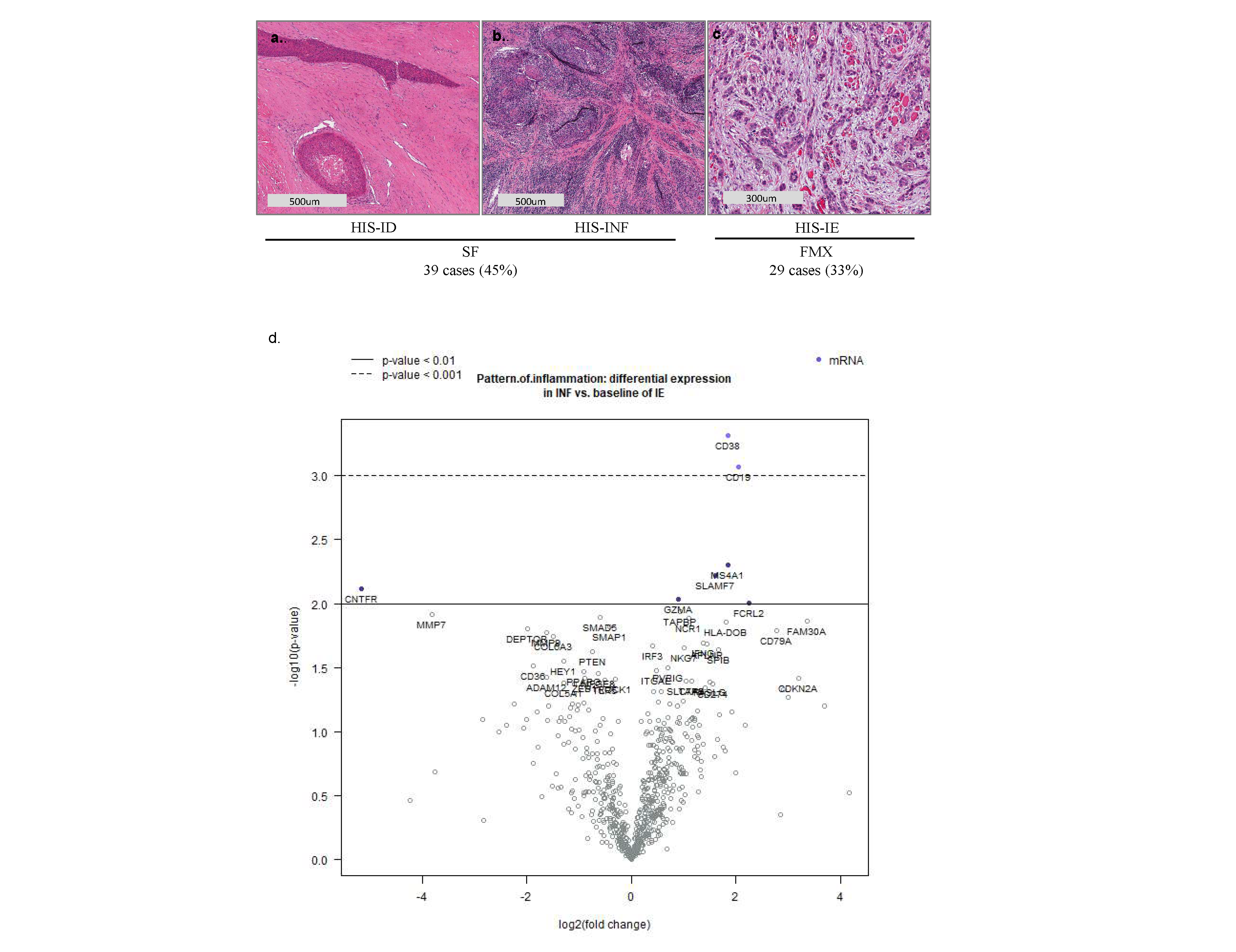

Supplement: Supplementary Image 1 — Stromal Density Characterizing Parameters. (A,B) SF was described as the presence of diffusely desmoplastic stroma in the areas of the tumor independent of the HIS subtype. (C) FMX stroma was the presence of loose, myxoid connective tissue adjacent to the tumor nests. (D) DGE analysis of HIS-INF relative to base line of HIS-IE. HIS, histologic inflammatory subtype; ID, immune deserted; INF, inflamed; IE, immune excluded; SF, stromal fibrosis; FMX, fibromyxoid; DGE, differential gene expression. [file Image1.tif]

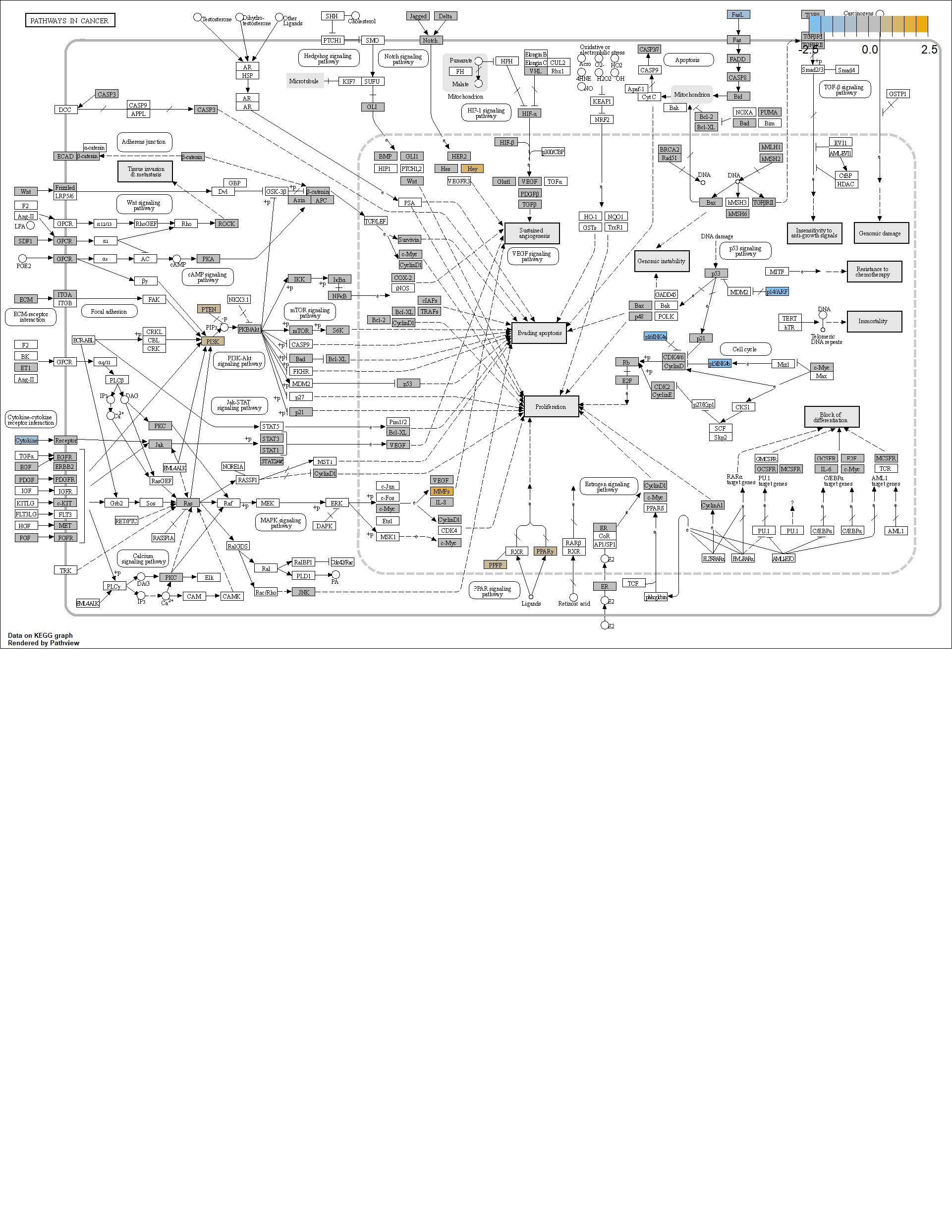

Supplement: Supplementary Image 2 — Kegg pathway analysis for “Pathways in Cancer” in HIS-IE vs. baseline of HIS-INF OSCC. [file Image2.tif]

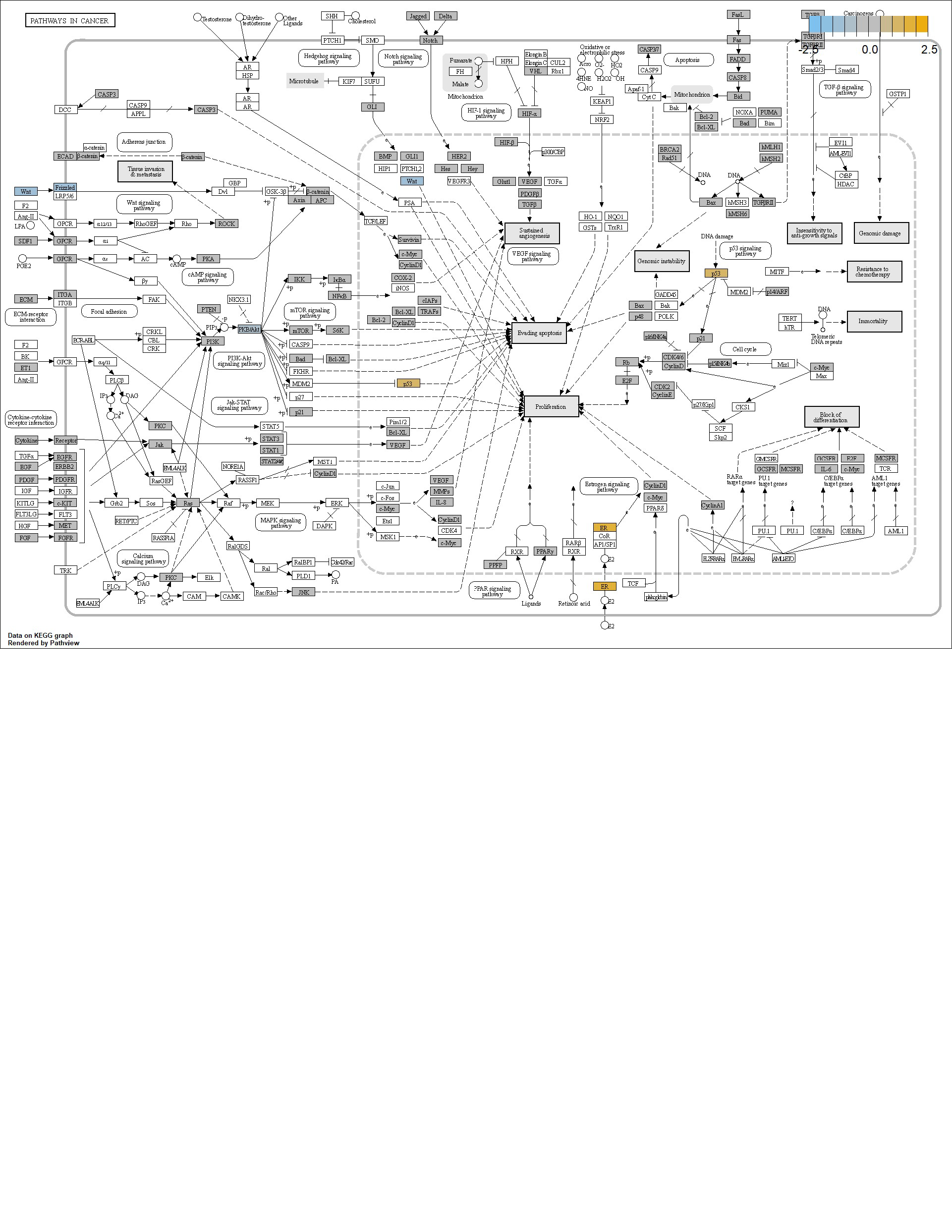

Supplement: Supplementary Image 3 — Kegg pathway analysis for “Pathways in cancer” using annotations of PTSI high vs. baseline of PTSI low. [file Image3.tif]

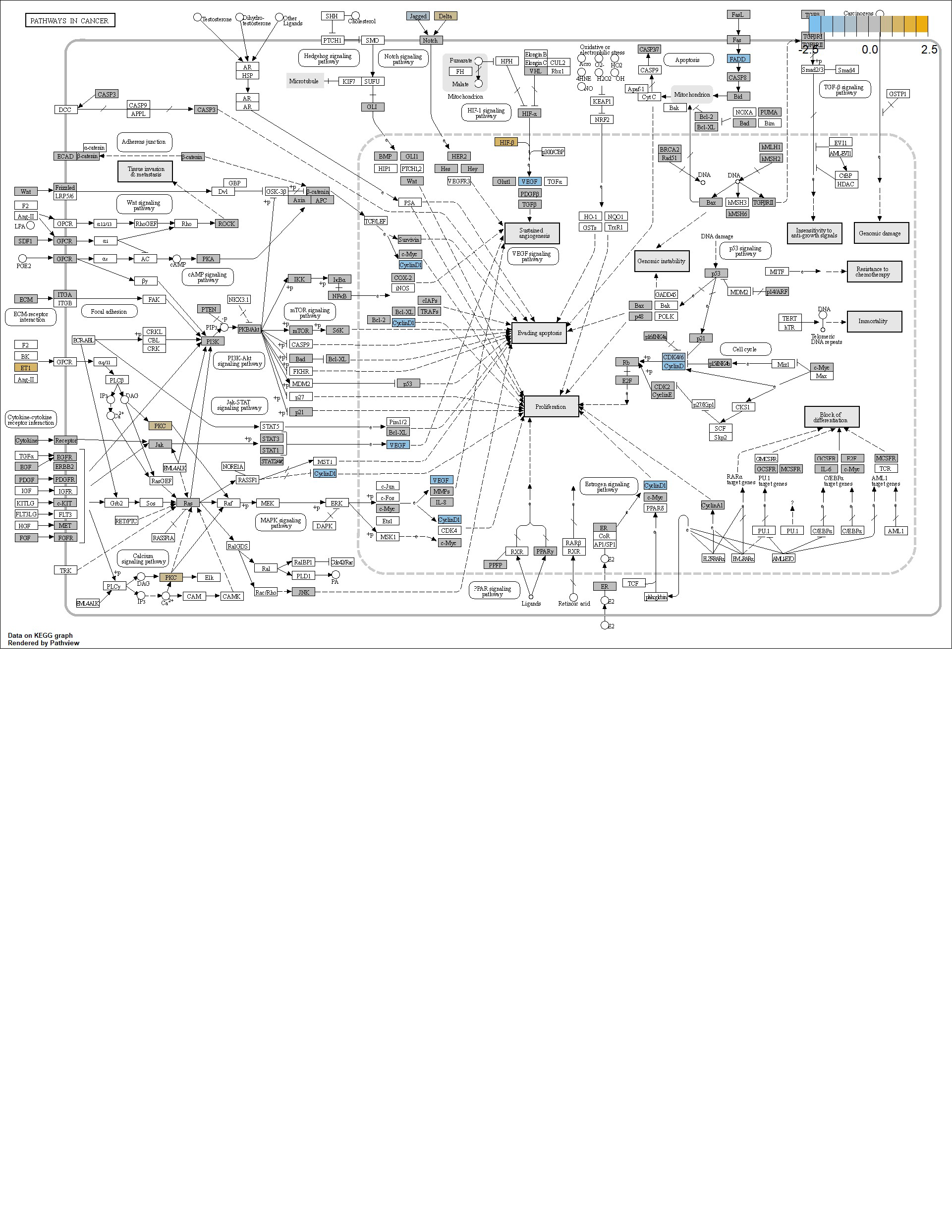

Supplement: Supplementary Image 4 — Kegg pathway analysis for “Pathways in cancer” signaling using annotations of PTSF high relative to baseline of PTSF low. [file Image4.tif]

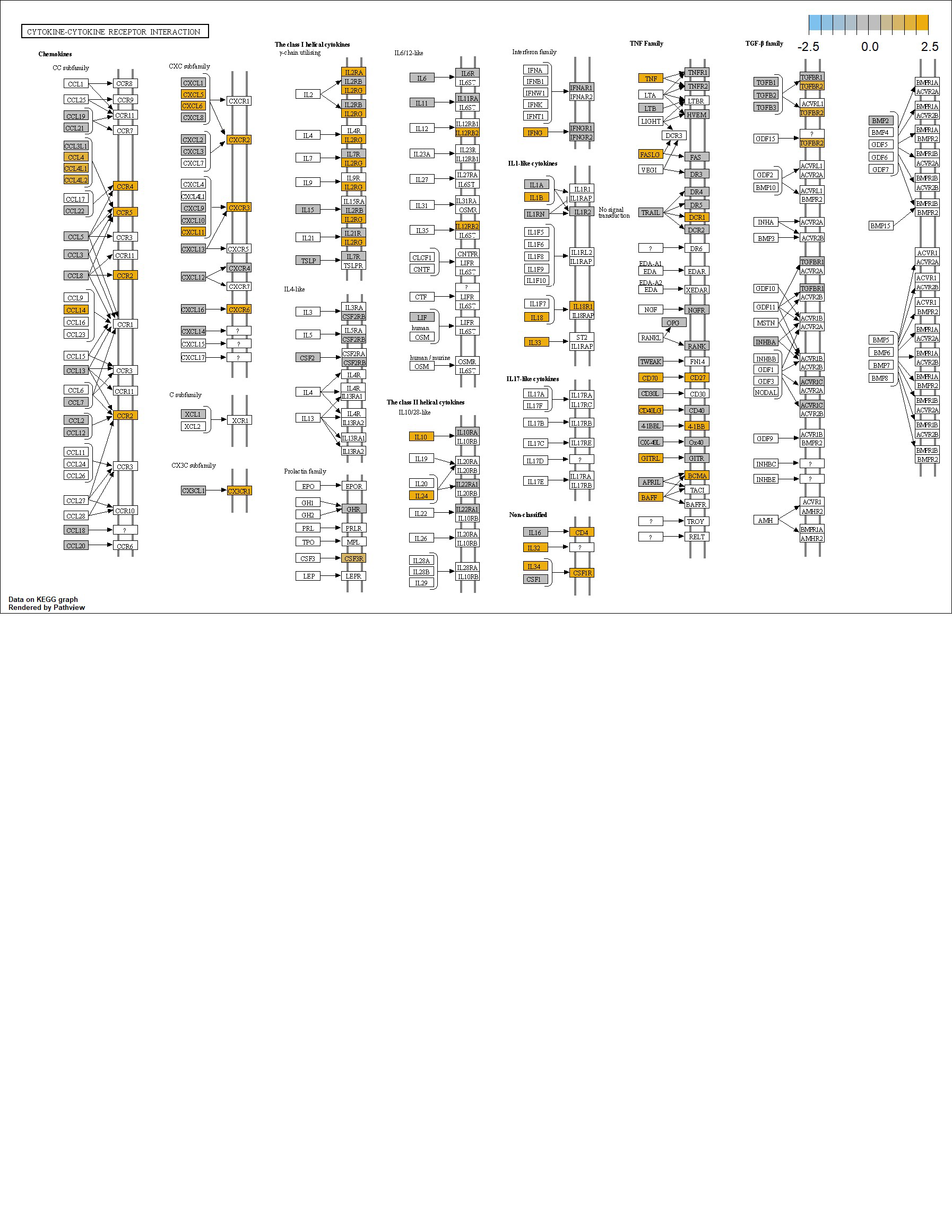

Supplement: Supplementary Image 5 — Kegg pathway analysis for cytokine/cytokine receptor signaling using non-smokers vs. baseline of smokers’ annotations. [file Image5.tif]
